# Inflammatory stimuli induce shedding of heparan sulfate from arterial but not venous porcine endothelial cells leading to differential proinflammatory and procoagulant responses

**DOI:** 10.1038/s41598-023-31396-z

**Published:** 2023-03-18

**Authors:** Anastasia Milusev, Alain Despont, Jane Shaw, Robert Rieben, Nicoletta Sorvillo

**Affiliations:** 1 Department for BioMedical Research (DBMR), University of Bern, Murtenstrasse 24, 3008 Bern, Switzerland; 2 Graduate School for Cellular and Biomedical Sciences (GCB), University of Bern, Bern, Switzerland

**Keywords:** Cell biology, Glycobiology, Coagulation system, Complement cascade, Inflammation

## Abstract

Endothelial dysfunction is an early event of vascular injury defined by a proinflammatory and procoagulant endothelial cell (EC) phenotype. Although endothelial glycocalyx disruption is associated with vascular damage, how various inflammatory stimuli affect the glycocalyx and whether arterial and venous cells respond differently is unknown. Using a 3D round-channel microfluidic system we investigated the endothelial glycocalyx, particularly heparan sulfate (HS), on porcine arterial and venous ECs. Heparan sulfate (HS)/glycocalyx expression was observed already under static conditions on venous ECs while it was flow-dependent on arterial cells. Furthermore, analysis of HS/glycocalyx response after stimulation with inflammatory cues revealed that venous, but not arterial ECs, are resistant to HS shedding. This finding was observed also on isolated porcine vessels. Persistence of HS on venous ECs prevented complement deposition and clot formation after stimulation with tumor necrosis factor α or lipopolysaccharide, whereas after xenogeneic activation no glycocalyx-mediated protection was observed. Contrarily, HS shedding on arterial cells, even without an inflammatory insult, was sufficient to induce a proinflammatory and procoagulant phenotype. Our data indicate that the dimorphic response of arterial and venous ECs is partially due to distinct HS/glycocalyx dynamics suggesting that arterial and venous thrombo-inflammatory disorders require targeted therapies.

## Introduction

Endothelial cells (ECs) comprise the inner lining of blood vessels and are crucial for regulating vascular homeostasis, as well as maintaining an anti-inflammatory and anticoagulant vessel phenotype^1^. In vascular disorders this homeostasis is disturbed due to endothelial dysfunction^2,3^ which leads to loss of vascular integrity and increased permeability^4^, reduced release of vasoactive agents such as nitric oxide (NO)^5^, as well as changes in thrombogenicity^6^ and in the expression of surface adhesion molecules which influence leukocyte interactions^7,8^. Endothelial dysfunction is strongly associated with shedding of the endothelial glycocalyx, a protective layer of proteins and sugars that covers the luminal surface of ECs^9^. The main components of the endothelial glycocalyx are proteoglycans, like syndecans, rich in glycosaminoglycan side chains, such as heparan sulfate (HS)^10^. Many regulatory plasma proteins, like antithrombin III (ATIII), C1-inhibitor or factor H have HS binding domains^11–13^ through which they interact with the glycocalyx. Binding of the complement regulatory protein factor H to cell-surfaces via glycosaminoglycans is crucial to protect against excessive complement activation^14^. On the other hand, binding of ATIII to the endothelial glycocalyx enhances its inhibitory activity towards the coagulation protein Factor XIa, thereby blocking activation of the coagulation cascade^15^. Furthermore, the glycocalyx has been suggested to form a nonadherent shield on the surface of ECs that inhibits excessive leukocyte adhesion, thereby reducing leukocyte transmigration and tissue inflammation^16^. Hence, the endothelial glycocalyx, in particular HS, plays a crucial role in regulating vascular homeostasis by maintaining an anti-inflammatory and anticoagulant environment. Loss of the endothelial glycocalyx has been described in many disorders^17^. For example, endothelial dysfunction concurrent with cardiovascular risk factors like hypertension, diabetes and obesity, is directly linked to loss of endothelial glycocalyx from the cell surface, increased thrombotic events and disease progression^18^.

Glycocalyx shedding also occurs during transplantation, where it is associated with poor graft survival and tissue rejection^19^. This is in part due to high levels of the proinflammatory cytokine TNFα^20,21^ which upregulates proteolytic enzymes, such as heparanase and matrix metalloproteinase 9 (MMP9), that cleave proteoglycans and HS from the cell surface causing disturbed vascular integrity and vascular rejection^22,23^. Recently, due to a lack of human organ donors, research has focused its attention on xenotransplantation, the transplantation of tissues or organs between two different species. Pigs are currently considered the most promising organ donor. In xenotransplantation, preformed recipient antibodies, targeting sugar residues on the surface of donor porcine ECs, induce glycocalyx shedding, complement deposition and endothelial dysfunction, ultimately culminating in organ rejection^24–26^.

Apart from xenotransplantation, another inflammatory condition in which endothelial glycocalyx shedding is considered a hallmark of disease is sepsis. There, the amount of glycocalyx components shed from ECs and measured in the plasma correlate with the development of disseminated intravascular coagulation^27^ and reduced patient survival^28^.

Although it has been shown that inflammatory conditions damage the glycocalyx and initiate or propagate disease, whether the glycocalyx on ECs from various vascular beds responds differently to inflammatory insults and if this has an impact on EC behavior is unknown. Pathophysiological differences in the hypercoagulable state of arterial and venous cells have been described^29^. ECs of the inferior vena cava compared to aortic ECs strongly upregulate the expression of adhesion molecules in response to the proinflammatory cytokine TNFα^30^. Whether this heterogeneity between ECs is due to differences in the glycocalyx pattern on the cell surface and whether this leads to a stronger endothelial dysfunction in veins compared to aorta is still unknown. We hypothesize that differences in glycocalyx dynamics influence the interaction of the endothelium with regulatory proteins leading to distinct responses in different vascular beds.

To determine whether glycocalyx dynamics are different for arterial and venous cells we compare the expression pattern of the glycocalyx on primary porcine arterial and venous ECs using a round-channel 3D microfluidic system. We focus on analyzing the role of HS, the main player in maintaining physiological glycocalyx functions^11^, in static or flow conditions, and upon stimulation with several inflammatory stimuli such as human serum, to mimic a xenotransplantation setting, TNFα and LPS. The interplay between the glycocalyx and endothelial dysfunction was analyzed by evaluating complement activation, leukocyte adhesion and activation of the coagulation cascade.

## Material and methods

### Isolation of primary porcine endothelial cells

Primary macrovascular ECs were isolated from porcine thoracic aorta and vena cava obtained from female and male landrace pigs aged 4–8 months after euthanasia. In accordance with the 3R principles, the vessels were obtained from animals used for terminal experiments by other research groups within the University of Bern. For these animals general anesthesia was provided through propofol (1–4 mg/kg) and ketamine (1 mg/kg), maintained with propofol (2–8 mg/kg/h) and fentanyl (5–30 g/kg/h) and additional analgesia was provided with ropivacaine (0.5%) and morphine (0.1 mg/kg). Death of the animal was defined as either the removal of the heart from the body or when the EEG signal remained flat for at least 60 s following administration of rocuronium and discontinuation of mechanical ventilation. Animal experiments and euthanasia were performed in accordance with Swiss Federal regulations and approved by the Cantonal Veterinary office, Berne, Switzerland. Arterial ECs were harvested mechanically from the lumen of the aorta using a humidified cotton swab, while venous ECs were harvested from the vena cava by enzymatic digestion using collagenase II (Worthington LS004174, 1.88U/ml in pure DMEM). Cells were grown in complete media (DMEM Glutamax, Gibco 21885-025) with 10% heat inactivated fetal bovine serum (FBS, Sigma F7542) and 1% Penicillin/streptomycin (P/S, Gibco 15140-122) supplemented with 1% endothelial growth medium 2 supplement mix (PromoCell C-39216) and monitored daily to avoid overgrowth of fibroblasts. Upon confluence, cells were phenotyped, cryopreserved and stored for future experiments. Cells were phenotyped by staining for EC markers CD31 and von Willebrand Factor as well as α smooth muscle actin to check purity. Only cultures expressing both EC markers and less than 10% α smooth muscle actin were used. Cells were used exclusively until passage four to avoid phenotypic drift. For all experiments, ECs from at least 2 different pig donors were used.

### Isolation of human and porcine peripheral blood mononuclear cells (PBMCs)

Peripheral blood mononuclear cells (PBMCs) were freshly isolated from human or porcine EDTA anticoagulated whole blood by density centrifugation. Human blood samples were obtained from healthy volunteers with informed consent and anonymized immediately after donation. The study protocol was approved by the local Ethics committee of the Canton of Bern and the experiments were performed in accordance with Swiss Federal regulations and guidelines of the University of Bern. For PBMC isolation, blood was centrifuged first to remove the plasma, which was then replaced with equal amounts of PBS-2%FBS. The diluted blood was layered onto Ficoll-Paque density gradient centrifugation medium (Cytivia 17144002) and PBMCs were isolated according to the manufacturer protocol. Subsequently, PBMCs were washed with PBS-2%FBS. Human PBMCs were used freshly after isolation while porcine PBMCs were frozen down in FBS with 10% DMSO (Dimethyl sulfoxide, Sigma D4540-100ML) for later usage.

### Culturing of endothelial cells in a 3D round-channel microfluidic system

Polydimethylsiloxane (PDMS) microfluidic chips containing 3D round-section channels with a diameter of 550 μm were prepared as described previously^31^. The microfluidic channels were coated with 50 μg/ml fibronectin (Merck FC010) and 100 μg/ml collagen II (Gibco A10644-01). Arterial and venous ECs were then seeded (1 million cells/ml) in flow media (DMEM with 10% FBS, 1% P/S, 4% dextran, Sigma 31390-100G and 1% bovine serum albumin, Sigma A7030-100G) and left to adhere overnight at 37 °C. Once confluent, each channel was connected to a peristaltic pump (Gilson minipuls 3). Using silicon tubing, flow media was drawn from a reservoir and passed through a bubble trap before reaching the cells. The peristaltic pump was adjusted to generate laminar shear stress of 2 dyn/cm^2^ or 12 dyn/cm^2^ (viscosity of the medium μ = 2.1 mPa s) for 72 h. Microfluidic chips were maintained in a humidified incubator at 37 °C and with 5% CO_2_. Media was changed every 24 h to supply fresh nutrients.

### Immunofluorescence

For microfluidic channels, cells were cultured for 72 h under flow or in static conditions and subsequently fixed with 4% formaldehyde (Sigma 252549-500ML stock solution 37%) for 20 min at room temperature. After blocking for one hour at room temperature with PBS-3%BSA, cells were incubated overnight at 4 °C with anti-heparan sulfate antibody (Amsbio 370-255-1, clone F58-10E4), wheat germ agglutinin (WGA) lectin (Sigma L4895-2MG) to stain N-Acetylglucosamine (GlcNAc) and sialic acid, anti-porcine CD31 antibody (R&D MAB33871) and/or polyclonal anti-human complement component C3b/c (DAKO A0062) diluted in PBS-1%BSA-0.05%Tween (Tween 20, AppliChem A4974,0250). Subsequently, samples were incubated for 1.5 h under agitation at room temperature with secondary antibodies: goat anti mouse IgM AlexaFluor568 (Invitrogen A21043) or goat anti mouse IgM AlexaFluor488 (Invitrogen A21042) for heparan sulfate, goat anti rat IgG AlexaFluor633 (Invitrogen A21094) or goat anti rat IgG AlexaFluor568 (Invitrogen A11077) for CD31 and/or goat anti rabbit IgG AlexaFluor633 (Invitrogen A21071) for C3b/c. All secondary antibodies were diluted in PBS-1%BSA-0.05%Tween. DAPI (Simga, 32670-20MG-F) was used for staining cell nuclei. Microfluidic chips were then washed and imaged using a 20 × objective on a Zeiss LSM710 AxioObserver or Zeiss LSM980 confocal microscope and analyzed using Image J (version 2.3.0/1.53q). GlcNAc/sialic acid and HS staining was analyzed by quantifying coverage according to a method adapted from Cheng et al.^32^. For quantification, the fluorescence threshold was adjusted to reduce background noise and the image was converted into a black and white mask. ImageJ was used to measure the percentage of white areas compared to the channel area and this percentage was equated to glycocalyx coverage, which was normalized to the number of cells per channel. At least three images were analyzed per sample. For chamber slides, used to characterize cultured ECs, the same procedure as described above was employed. Slides were then mounted using ProLong gold antifade reagent (Invitrogen P36934) and acquired with a fluorescent microscope at 20× magnification (Leica DMI4000B).

En face vessels were prepared from freshly isolated porcine thoracic aorta and vena cava which were cut into 1cm^2^ pieces and fixed with 4% formaldehyde for 20 min at room temperature. Vessel pieces were then blocked and stained for von Willebrand Factor (DAKO A0082), CD31 and glycocalyx components as described above. Images were acquired with a confocal microscope (Zeiss AxioObserver LSM710).

To obtain vessel sections, rings of freshly isolated porcine thoracic aorta and vena cava were embedded in TissueTek O.C.T compound (Sakura 4583). 5 μm thick sections were fixed with cold 100% acetone (AppliChem 141007.1211) and incubated for 30 min with 20% NHS diluted in TBS to induce HS shedding and complement deposition. Control sections were incubated with TBS only. Cryosections were blocked with TBS-3%BSA, stained and mounted as described above. Images were acquired with a confocal microscope at 20× magnification (Zeiss LSM980). To image the glycocalyx on live unfixed cells, microfluidic channels were perfused for 72 h (2 dyn/cm^2^ or 12 dyn/cm^2^) and then stained during 30 min under flow with WGA-Lectin and Hoechst nuclear stain using the dilutions described above in flow media without FBS. Channels were immediately imaged using a confocal microscope at 20× magnification (Zeiss LSM980).

### Complement deposition on endothelial cell surface

After 72 h of high shear stress (12 dyn/cm^2^) cells were left untreated or incubated under flow with either 10% normal human serum (collected from healthy volunteers) for 2 h, 100 ng/ml recombinant human tumor necrosis factor α (TNFα, R&D 210-TA), 100 μg/ml lipopolysaccharide (LPS, Sigma L4391) or 5 U/ml heparinase I + III (Sigma H3917-100UN) for 4 h at 37 °C in serum-free flow media. To evaluate complement deposition on TNFα, LPS and heparinase I + III stimulated cells, channels were additionally perfused with 10% porcine serum during the last 2 h of activation. After fixation, samples were stained using an anti-complement C3b/c antibody as described above.

### PBMC – EC adhesion assay

Cells were seeded in 3D round-section microfluidic channels and perfused for 72 h under 12 dyn/cm^2^ shear stress conditions after which they were treated with normal human serum, recombinant human TNFα, LPS or heparinase I + III as described above. Nuclei of ECs within the channels were stained under flow with Hoechst 3342 nuclear staining (Tocris 23491-52-3). Next, human or porcine isolated PBMCs were labeled with CFSE (ThermoFisher C34554) according to manufacturer’s instructions and re-suspended in flow media without dextran. Each channel was perfused at a shear stress of 0.2 dyn/cm^2^ with 1 million/ml labeled PBMCs while pictures were acquired every 3 s for 20 min. Video files with a frame rate of 3 frames per second were created for analysis. PBMC adhesion was defined as the number of cells immobilized for at least 3 s (i.e., one frame). Cells were counted manually for each time-lapse recording. Channels were fixed after PBMC perfusion and stained for HS as described above.

### Microfluidic clotting assay

Cells were seeded in 3D round-section microfluidic channels as described above and subsequently perfused at a shear stress of 12 dyn/cm^2^ with recalcified human (from healthy anonymous volunteers) or porcine (Merck P2891) citrate plasma spiked with 15 μg/ml AF488 labeled human fibrinogen (Thermofisher F13191). Fibrinogen spiked plasma was recalcified by addition of 25 mM (human plasma) or 13 mM (porcine plasma) CaCl_2_ (Sigma 10043-52-4) immediately before imaging. During perfusion cells were imaged every 5 s for up to 15 min using a confocal microscope (Zeiss LSM980). The experiment was terminated when complete occlusion of the channel occurred or when occlusion of the channel caused an increase of pressure within the microfluidic system leading to complete cell detachment from the channel surface. Time to occlusion was determined from video files with a frame rate of 3 frames per second.

### Statistical analysis

All data sets were analyzed using GraphPad Prism 9 software, and *p* < 0.05 was considered significant. One-way ANOVA followed by multiple comparisons using pairwise Tukey t-test was performed for data sets with more than 2 groups. For complement deposition, PBCM adhesion and clotting two-way ANOVA was performed followed by multiple comparisons using Tukey and Bonferroni post-hoc analysis. Experiments were performed with a minimum of three biological replicates.

## Results

### Laminar shear stress influences heparan sulfate expression and distribution on the cell surface of arterial and venous endothelial cells

**Figure 1:**
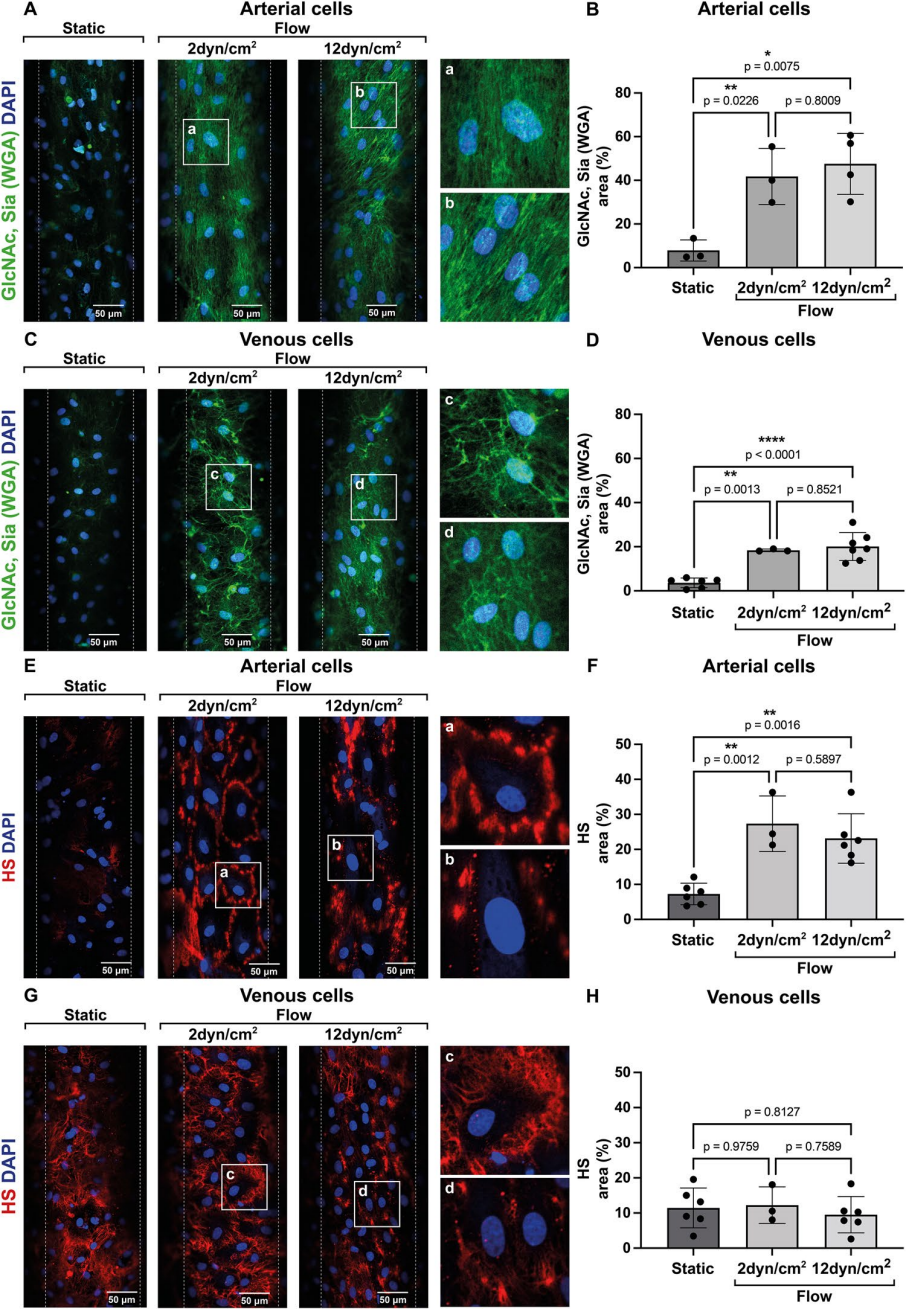
Influence of shear stress on glycocalyx/N-Acetylglucosamine, sialic acid and heparan sulfate coverage on arterial and venous endothelial cells. Representative images of microfluidic channels containing (**A**,**E**) arterial or (**C**,**G**) venous porcine endothelial cells cultured under static conditions, at low (2 dyn/cm^2^) or high shear stress (12 dyn/cm^2^). Cells were stained (**A**,**C**) for N-Acetylglucosamine (GlcNAc) and sialic acid (Sia) in green or (**E**,**G**) for heparan sulfate (HS) in red. Nuclei are shown in blue (DAPI). All images were acquired with a Zeiss LSM710 confocal microscope. Scale bar: 50 μm. (**B**,**D**,**F**,**H**) Coverage of glycocalyx components (GlcNAc/Sia or HS) was calculated for each image (4 images/condition/experiment) as percentage of area positive for (**B**,**D**) GlcNAc and Sia or (**F**,**H**) for HS and, was normalized to the total number of cells/image. Data are from three or more independent experiments and 2–3 different porcine ECs donors. One-way ANOVA with multiple comparisons was used for statistical analysis.

To determine how shear stress affects the expression of different glycocalyx components and investigate whether the glycocalyx could account for the diverse behavior of arterial and venous ECs during inflammatory disorders we analyzed coverage of the glycocalyx on the EC surface by immunofluorescence. Venous and arterial cells were grown for 72 h under static or laminar shear stress conditions (12 dyn/cm^2^ and 2 dyn/cm^2^) in a 3D round-channel microfluidic system^31^. These shear stress conditions were chosen in accordance with physiological shear stress values for aorta and vena cava reported from in vivo data ^33^. Cells were stained using wheat germ agglutinin (WGA) lectin, which binds to N-Acetylglucosamine (GlcNAc) and sialic acid (Sia) residues present within the endothelial glycocalyx. WGA staining has been widely used to specifically label, visualize, and quantify glycocalyx on vascular endothelium^34,35^. As shown in Fig. 1A–D, WGA staining (GlcNAc, Sia) was mainly observed upon laminar shear stress on both arterial and venous ECs. On the other hand, HS, the major glycocalyx component expressed by ECs responsible for maintaining physiological anti-inflammatory and anticoagulant properties, was flow-dependent on arterial cells (Fig. 1E,F), while surprisingly, HS was already observed under static conditions on the surface of venous ECs (Fig. 1G,H). This indicates that glycocalyx dynamics differ between arterial and venous cells. Furthermore, changes in shear stress influenced HS distribution. Under low shear conditions (2 dyn/cm^2^) HS was located along the junctional regions of ECs, while when high shear stress was applied, HS clustered to distinct areas of the cell membrane (Fig. 1E,G). Clustering of proteoglycans containing HS, like syndecan-4, has been shown to occur at specific membrane lipid raft regions and is important for vesicular trafficking and signal transduction^36^.

To ensure that ECs in vitro have a similar phenotype as ECs in vivo, freshly isolated porcine thoracic aorta and vena cava were stained *en face* with EC markers, such as CD31 and vWF (von Willebrand Factor), and for the glycocalyx, with both WGA lectin and anti-HS antibody. As shown in Supplementary Fig. S1, ECs grown in vitro show a similar phenotype to ECs in vivo in blood vessels.

### Venous endothelial cells are resistant to heparan sulfate shedding after xenogeneic activation

Alterations in the glycocalyx and shedding of HS causes loss of protein binding and is observed during ischemia reperfusion injury and transplantation. Currently, due to global organ shortage, the transplantation of organs between two different species is highly considered^37^.

**Figure 2:**
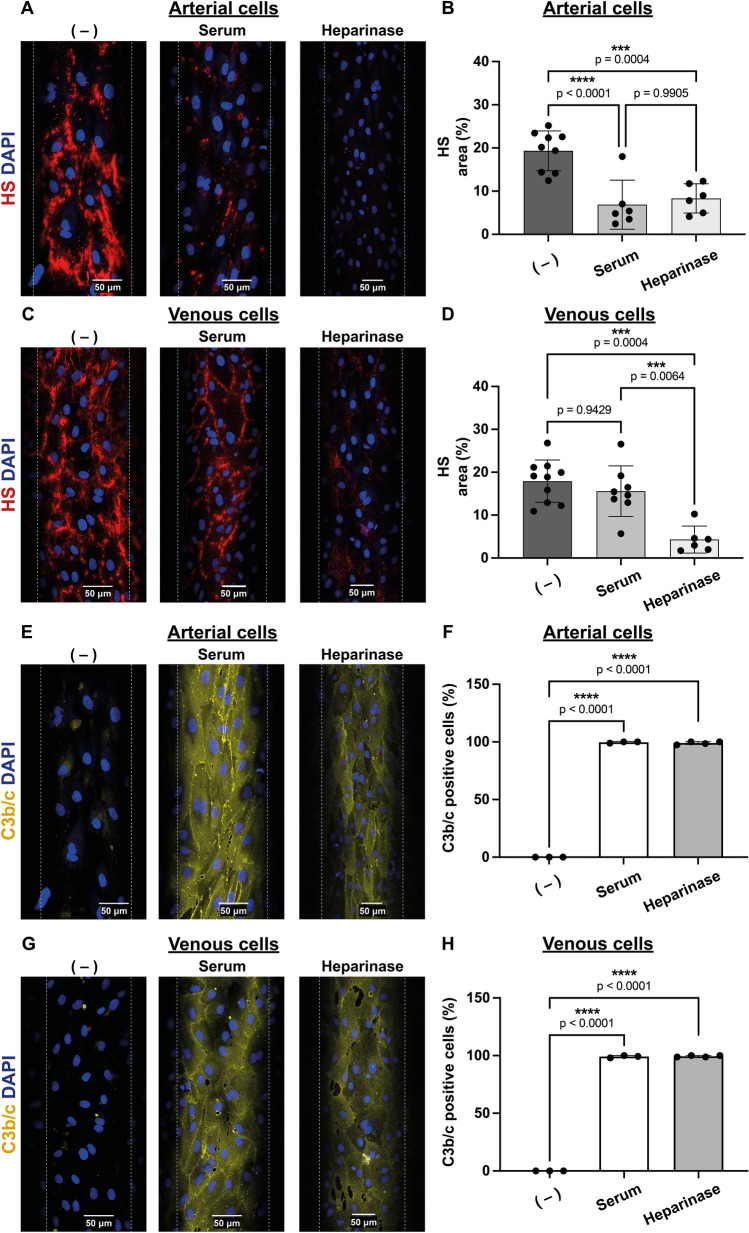
Resistance of heparan sulfate shedding on xenogeneic activated venous cells does not prevent C3b/c deposition. (**A**,**C**) Representative images of heparan sulfate (HS) on the cell surface of cells left untreated (−), perfused with 10% normal human serum (Serum) or 5 U/ml heparinase (Heparinase). HS is shown in red and nuclei in blue (DAPI). Scale bar: 50 μm (**B**,**D**) Shedding of HS was quantified for each image (4 images/condition/experiment) as the percentage of area positive for HS and normalized for the total number of cells/image. (**E**,**G**) Representative images of complement C3b/c deposition on the cell surface of cells left untreated (−), perfused with 10% normal human serum (Serum) or 5 U/ml heparinase (Heparinase). C3b/c is shown in yellow and nuclei in blue (DAPI). Scale bar: 50 μm (**F**,**H**) C3b/c deposition was measured as the percentage of C3b/c positive cells/total number of cells/image (4 images/condition/experiment). Data was quantified with Fiji software. All images were acquired with a Zeiss LSM980 confocal microscope. Data are from three or more independent experiments and 2–3 different porcine ECs donors. One-way ANOVA with multiple comparisons was used for statistical analysis.

Therefore, to determine whether the difference in glycocalyx dynamics between arterial and venous cells would induce distinct responses during an inflammatory condition like xenotransplantation, porcine arterial and venous ECs were perfused with normal human serum (NHS) to mimic in vitro a xenotransplantation environment. It has been shown that binding of pre-formed xenoreactive antibodies to xenoantigens expressed on the EC surface, such as α-galactose^24^ induces shedding of the EC glycocalyx^38^ leading to graft rejection. We observed shedding of HS from the surface of arterial ECs stimulated with NHS (Fig. 2A,B; (−) and serum). This correlated with increased expression of E-selectin (Supplementary Fig. S2A; (−) and serum), an important adhesion molecule responsible in part for the interaction of ECs with leukocytes. Surprisingly, stimulation of venous ECs with NHS did not induce HS shedding; a 16–18% HS coverage was observed on both activated and non-activated ECs (Fig. 2C,D; (−) and serum). However, as for arterial ECs, E-selectin expression was increased after incubation with NHS (Supplementary Fig. S2B). To ensure that HS can indeed be shed from the surface of venous cells, ECs were perfused with heparinase I + III, an enzyme that specifically cleaves HS. As observed in Fig. 2A,C, incubation with heparinase removes approximately 50% (Fig. 2B) and 70% (Fig. 2D) of HS from arterial and venous cells, respectively. Other glycocalyx components such as GlcNAc and sialic acid were not affected by NHS (Supplementary Fig. S3A, B) nor, as expected, by heparinase treatment (Supplementary Fig. S3C).

To confirm that our observations are not only restricted to cultured ECs, cryosections of freshly isolated porcine thoracic aorta and vena cava were treated ex vivo with NHS and analyzed for HS coverage. Again, shedding of HS from the artery but not from the vein was observed (Supplementary Fig. S4). Furthermore, to exclude possible artifacts in glycocalyx coverage due to fixation, GlcNAc/Sia staining was performed on live, unfixed cells. As shown in Supplementary Fig. S5, coverage of GlcNAc/Sia is comparable on both live and fixed arterial and venous ECs under low and high shear stress conditions. In addition, to ensure that the high shear stress used in the experimental set-up was not interfering with HS shedding from venous ECs, which normally require a lower shear environment, NHS perfusion was conducted under venous shear stress conditions (2 dyn/cm^2^). Once more, HS was only shed from the surface of arterial cells (Supplementary Fig. S6A–D).

### Heparan sulfate does not regulate the anti-inflammatory and anticoagulant properties of venous endothelial cells in a xenotransplantation setting

Shedding of HS has been linked to a proinflammatory EC phenotype prone to complement deposition, leukocyte adhesion and coagulation.

Complement deposition on both arterial and venous cells was evaluated by immunofluorescence after perfusion of cells with NHS at a shear stress of 12 dyn/cm^2^. The choice of a higher shear stress was based on the observation that shear can enhance complement receptor expression on ECs and allow for a better visualization of complement deposition^39^. All three pathways of the activated complement cascade merge at the level of C3, an essential component of the complement system where signal amplification takes place^40^. Therefore, deposition of C3b/c, the product of C3 cleavage by complement C3 convertase enzymes, was used as a marker for complement activation. Interestingly, although shedding of the endothelial glycocalyx was observed only from NHS activated arterial cells (Fig. 2A–D), complement C3b/c deposition was observed on both cell types (Fig. 2E–H), indicating that the persistence of HS on venous cells does not protect against complement deposition. To determine whether removal of HS would enhance complement deposition, cells were treated with heparinase. Binding of C3b/c to arterial and venous cells did not increase compared to NHS stimulated cells (Fig. 2E–H), confirming that HS on venous cells does not play a role in regulating complement deposition in a xenotransplantation set up.

To investigate whether complement deposition on the cell surface promotes shedding of HS, channels were perfused with heat inactivated NHS (HI serum). Heat blocks activation of the complement system, therefore only a minimal deposition of C3b/c is expected. Incubation of both arterial and venous cells with heat inactivated NHS did not induce shedding of HS (Supplementary Fig. S7A–D) and C3b/c deposition (Supplementary Fig. S7E–H). This demonstrates that HS shedding from arterial cells could be significantly reduced by inactivating the complement system, suggesting that complement deposition on arterial cells correlates with HS shedding.

**Figure 3:**
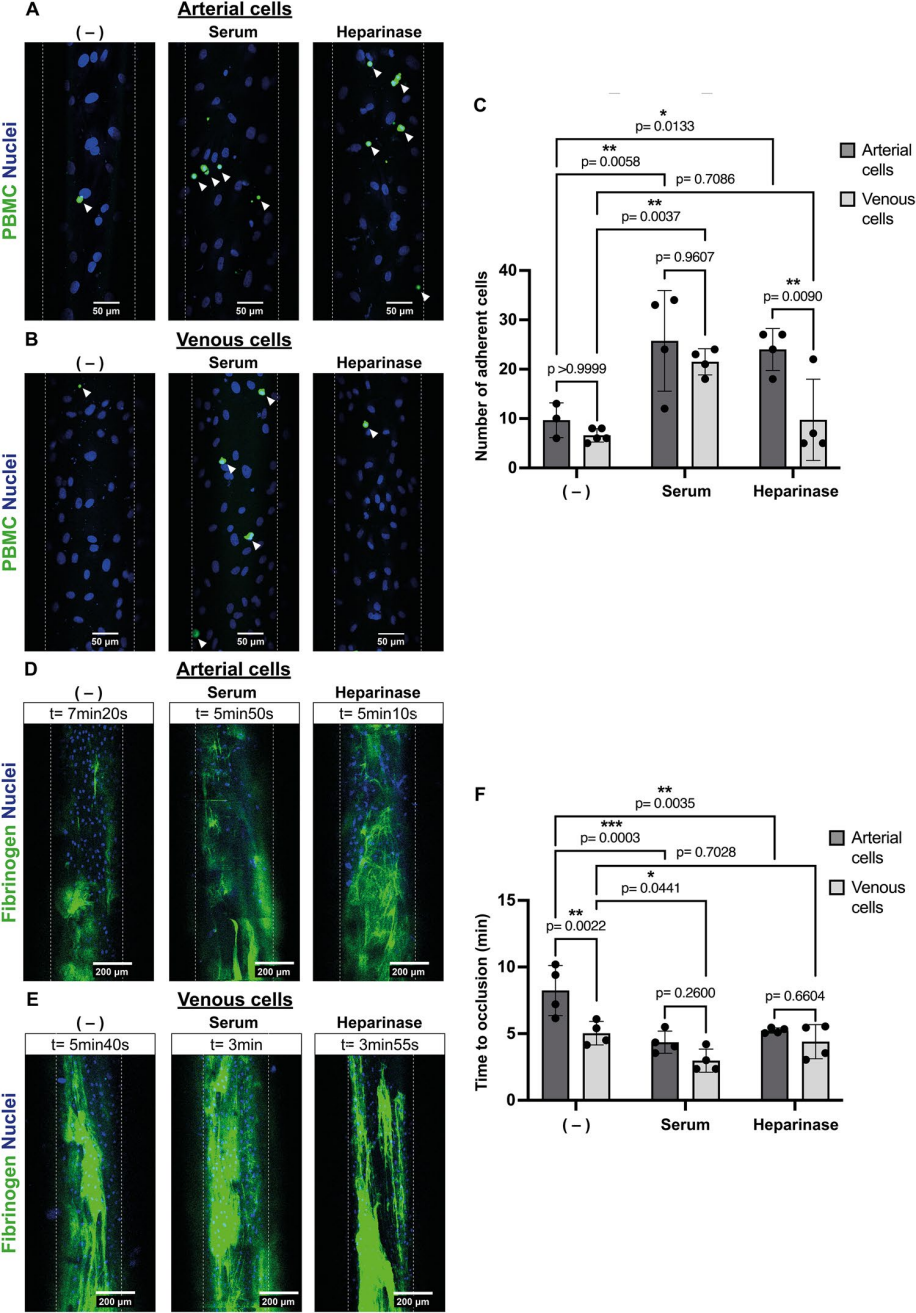
Persistence of heparan sulfate on venous cells after xenogeneic activation fails to prevent leukocyte adhesion and clot formation. (**A**,**B**) Representative images of 3 time-lapse recordings of binding of human peripheral blood mononuclear cells (PBMCs; shown in green) to (**A**) arterial and (**B**) venous porcine endothelial cells left untreated (−) or perfused with either 10% normal human serum (Serum) or 5 U/ml heparinase (Heparinase). Nuclei of cells are shown in blue (Hoechst). Scale bar: 50 μm. (**C**) Adherent PBMCs were quantified from 20 min time-lapse recordings of four independent experiments by counting cells immobilized for ≧ 3 s. (**D**,**E**) Representative images of fibrin clots (green) forming on (**D**) arterial or (**E**) venous porcine ECs. Cells were left untreated (−) or perfused with either 10% normal human serum (Serum) or 5 U/ml heparinase (Heparinase) prior to addition of recalcified citrated human plasma spiked with AlexaFluor488 labeled human fibrinogen (green). Scale bar: 200 μm. (**F**) Time to occlusion was determined as complete channel occlusion or cell detachment and quantified from time-lapse recordings of plasma perfused channels. Data are from four independent experiments, 2–3 different porcine ECs donors and were analyzed using Fiji software. Two-way ANOVA with Tukey and Bonferroni post-hoc test was used for statistical analysis.

Next, we evaluated binding of human PBMCs to xenogeneic activated porcine ECs. Adhesion of PBMCs was quantified as the number of leukocytes that bind to the cell surface for at least 3 s. A similar amount of PBMC binding to arterial and venous cells was detected (Fig. 3A–C). As shown in Supplementary Fig. S2, E-selectin is expressed on both arterial and venous cells after perfusion with NHS.

To determine whether HS shedding in the absence of an inflammatory stimulus causes leukocyte binding, ECs were treated with heparinase. Heparinase does not activate ECs, in fact, no E-selectin was expressed on the cell surface of either cell type (Supplementary Fig. S2). Unexpectedly, shedding of HS from heparinase treated arterial but not venous cells was sufficient to induce adhesion of human PBMCs (Fig. 3C). This indicates that E-selectin expression is essential for leukocyte binding to venous ECs, while HS influences PBMC adhesion to arterial ECs.

We then analyzed the impact of HS shedding on clot formation by perfusing both arterial and venous cells with recalcified human plasma spiked with fluorescently labeled human fibrinogen. As shown in Fig. 3D–F, time to clot formation of both arterial and venous ECs activated with NHS was reduced by approximately 50% and 40%, respectively, compared to untreated cells (Fig. 3F), implying that intact HS on venous cells does not prevent clotting. Interestingly, untreated arterial cells had a time to clot formation of 8 min whereas channels containing venous cells occluded already after 5 min (Fig. 3F). This indicates that there is a fundamental difference in the clotting time between these two cell types.

To further investigate the role of HS in coagulation, cells were treated with heparinase prior to perfusion with fibrinogen-spiked human plasma. As shown in Fig. 3D–F, while HS shedding reduced the time to occlusion for channels seeded with arterial ECs, no effect was observed on venous cells. This suggests that removal of HS from arterial cells is sufficient to induce a procoagulant phenotype.

### Unshed heparan sulfate on venous endothelial cells after stimulation with TNFα and LPS prevents complement deposition

**Figure 4:**
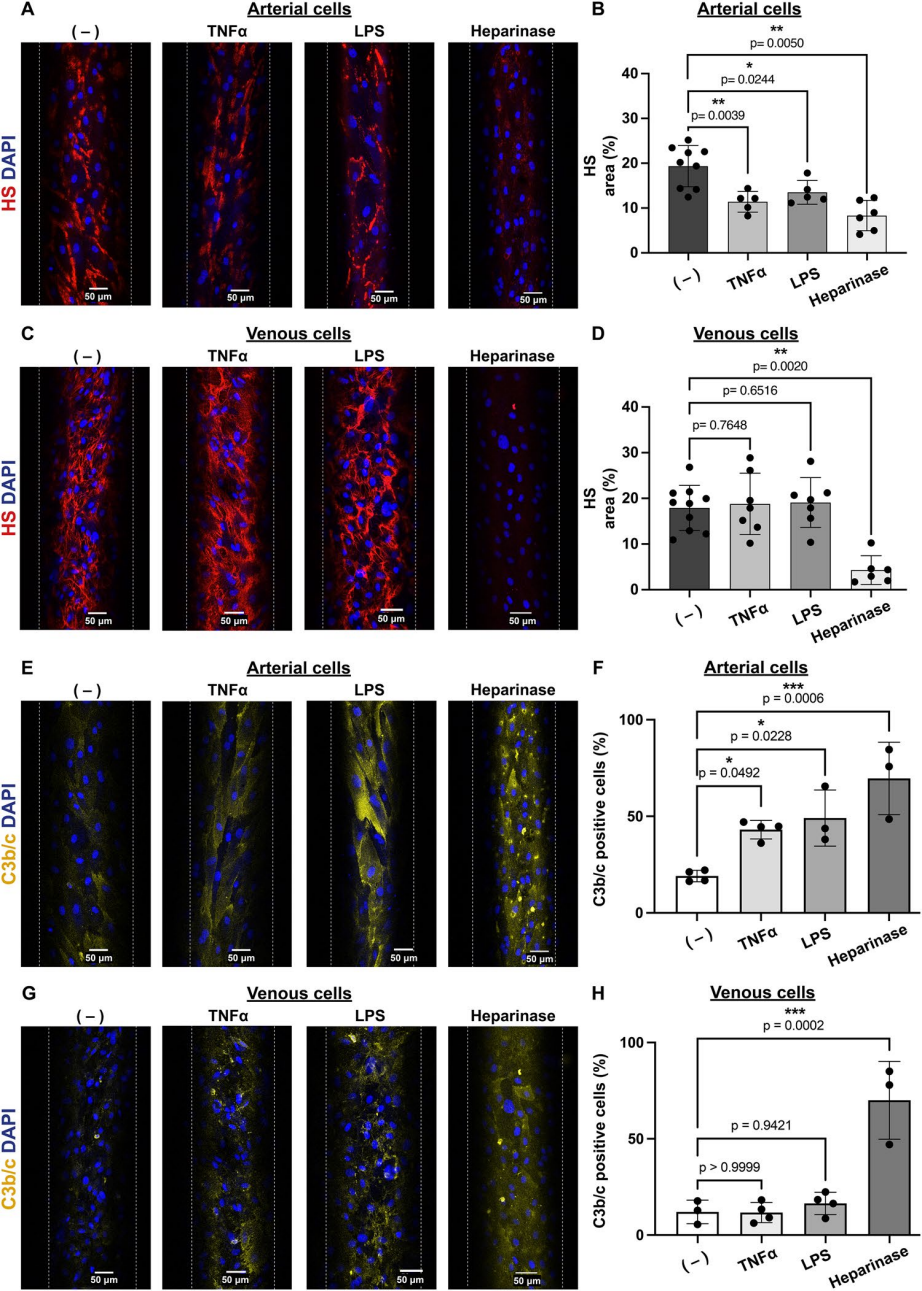
Unscathed heparan sulfate on venous cells after stimulation with TNFα or LPS prevents complement deposition. (**A**,**C**) Representative images of cells left untreated (−) or perfused with either 100 ng/ml TNFα, 100 μg/ml LPS or 5 U/ml heparinase (Heparinase). Heparan sulfate (HS) is shown in red, and nuclei in blue (DAPI). Scale bar: 50 μm. (**B**,**D**) Shedding of HS was quantified for each image (4 images/condition/experiment) as the percentage of area positive for HS and normalized for the total number of cells/image. (**E**,**G**) Representative images of complement C3b/c on the cell surface of cells left untreated (−) or perfused with either 100 ng/ml TNFα, 100 μg/ml LPS or 5 U/ml heparinase (Heparinase). C3b/c is shown in yellow and nuclei in blue (DAPI). Scale bar: 50 μm (**F**,**H**) C3b/c deposition was measured as the percentage of C3b/c positive cells/total number of cells/image (4 images/condition/experiment). Two-way ANOVA with Tukey and Bonferroni post-hoc test was used for statistical analysis. Data are from three or more independent experiments, 2–3 different porcine ECs donors and were analyzed using Fiji software.

To determine whether venous cells are resistant to HS shedding also under different proinflammatory conditions, we stimulated ECs with TNFα and LPS and measured HS coverage as described above. Shedding of HS was observed for TNFα and LPS activated arterial ECs (Fig. 4A,B), but again not for activated venous ECs (Fig. 4C,D). In fact, HS was reduced on the cell surface of TNFα and LPS treated arterial cells by 41% and 30% respectively, compared to control (Fig. 4B), whereas it was unchanged on the surface of venous ECs (Fig. 4D).

To evaluate if persistence of HS on the EC surface would allow cells to maintain an anti-inflammatory phenotype, ECs were perfused with normal porcine serum (NPS), and complement deposition was evaluated. As observed in Fig. 4E–H, a small amount of C3b/c was detected on the surface of unstimulated ECs. This is likely caused by the immunological mismatch between the ECs and NPS obtained from different donors. Stimulation of arterial cells with TNFα or LPS increased C3b/c deposition by approximately 25% and 30%, respectively (Fig. 4F) confirming that shedding of HS from arterial cells correlates with a loss of anti-inflammatory properties. On the other hand, perfusion of TNFα or LPS activated venous cells with NPS did not lead to complement deposition (Fig. 4H), suggesting that in these inflammatory conditions, HS on venous cells is protective. To further confirm the protective role of HS, both arterial and venous ECs were perfused with heparinase. As shown in Fig. 4, enzymatic removal of HS by heparinase (Fig. 4A–C) from the cell surface was sufficient to induce deposition of C3b/c on both arterial and venous ECs (Fig. 4F,H).

### Persistence of heparan sulfate on venous endothelial cells after stimulation with TNFα and LPS does not prevent PBMC adhesion but protects against clotting

**Figure 5:**
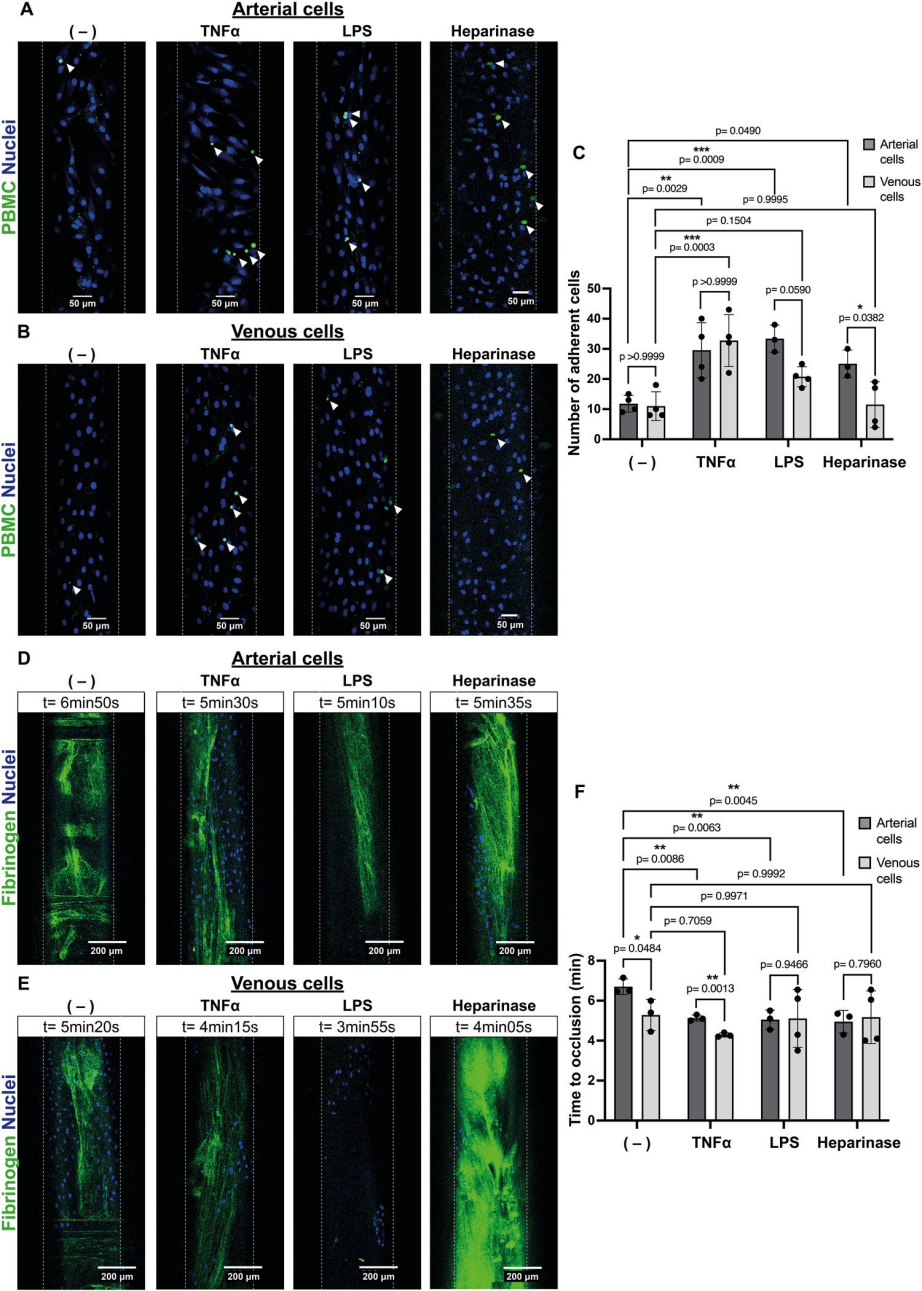
Intact heparan sulfate on TNFα or LPS activated venous endothelial cells does not impede leukocyte adhesion but protects against clot formation. (**A**,**B**) Representative images of three time-lapse recordings of binding of porcine peripheral blood mononuclear cells (PBMCs; shown in green) to (**A**) arterial and (**B**) venous porcine endothelial cells left untreated (−) or perfused with either 100 ng/ml TNFα, 100 μg/ml LPS or 5 U/ml heparinase (Heparinase). Nuclei of cells are shown in blue (Hoechst). Scale bar: 50 μm. (**C**) Adherent PBMCs were quantified from 20 min time-lapse recordings of four independent experiments by counting cells immobilized for ≧ 3 s. (**D**,**E**) Representative images of fibrin clots (green) forming on (**D**) arterial or (**E**) venous porcine endothelial cells. Cells were left untreated (−) or perfused with either 100 ng/ml TNFα, 100 μg/ml LPS or 5 U/ml heparinase (Heparinase). Nuclei of cells are shown in blue (Hoechst). Scale bar: 200 μm. (**F**) Time to occlusion was determined as complete channel occlusion or cell detachment and quantified from time-lapse recordings of plasma perfused channels spiked with AlexaFluor488 labeled fibrinogen (green). Data are from four independent experiments, 2–3 different porcine ECs donors and were analyzed using Fiji software. (**C**) Two-way ANOVA with Tukey and Bonferroni post-hoc test or (**F**) one-way ANOVA with multiple comparisons was used for statistical analysis.

Next, we investigated whether the observed difference in HS dynamics between TNFα and LPS activated arterial and venous cells would impact their interaction with leukocytes. For this, TNFα or LPS activated arterial and venous ECs were perfused with fluorescently labeled porcine PBMCs. Increased PBMC binding was observed for both activated arterial and venous ECs (Fig. 5A–C). This correlated with the cell surface expression of E-selectin (Supplementary Fig. S2B). Together, this suggests that although HS is not shed from the surface of venous ECs (Fig. 2C,D), the increased expression of E-selectin is sufficient to allow PBMC adhesion. Indeed, shedding of HS by heparinase prior to PBMC perfusion on venous cells did not increase PBMC adhesion, whereas on arterial cells, removal of HS without prior activation was sufficient to induce PBMC binding (Fig. 5A,C).

Since persistence of HS on venous cells after stimulation with TNFα or LPS was able to protect against complement deposition, we investigated if clot formation would also be impaired. For this, venous and arterial cells were left untreated or activated with TNFα, LPS or heparinase and then perfused with recalcified normal pooled porcine plasma spiked with AlexaFluor488 fluorescently labeled fibrinogen. As shown in Fig. 5D–F, time to clot formation was reduced only for arterial but not for venous ECs (Fig. 5F). This indicates that HS on venous cells participates in maintaining an anticoagulant state. However, since heparinase does not lead to increased clot formation, these data suggest that other hits, such as damage to the endothelial layer or induction of procoagulant tissue factor expression, are necessary to promote venous thrombosis.

## Discussion

Endothelial glycocalyx shedding has been observed in many inflammatory conditions^18,22,23,27^, however, the effect of inflammation on arterial or venous cells and the outcome in terms of glycocalyx disruption is largely unknown. Here, we show that the glycocalyx on venous and arterial ECs responds differently to both shear stress and inflammatory cues. Shear stress is known to influence the glycocalyx development^41–45^. Reduced or disturbed flow on arterial cells is not only linked to a pro-atherogenic phenotype favoring atherosclerosis^46–48^, but also to reduced glycocalyx thickness^49^ that affects glycocalyx function causing inflammation and coagulation. We found that there are distinct glycocalyx dynamics, in particular for HS, on porcine arterial and venous ECs under different shear stress conditions. HS on arterial cells is only expressed under flow, whereas on venous cells it is present even under static conditions (Fig. 1). We have investigated the expression of glycosaminoglycan (GAG) side chains, however, it is still unclear how shear stress influences core proteins which are the anchor points for different GAG side chains. It is possible that core proteins expressed under static conditions are decorated by a different set of N-substituted (stained by anti-HS antibody) and N-unsubstituted (stained by WGA-Lectin) disaccharide units than under flow, explaining the presence of HS but low expression of GlcNAc/Sia on venous ECs under static conditions. Further research is needed to investigate core proteins in addition to specific GAG side chains under static and flow conditions. Moreover, the distribution of HS on the cell surface is influenced by changes in shear stress. HS clusters to specific cell membrane regions under high shear, while it distributes along cell junctions under low shear conditions (Fig. 1). Redistribution of glycocalyx components like syndecan-4 to lipid rafts was observed by Zeng et al.^41^ and was suggested to allow aggregation of signaling molecules and induce signal transduction. Interestingly, HS clustering to lipid rich junctional membrane regions was also shown to facilitate SARS-CoV 2 virus entry into host cells^50^. Therefore, variations in HS distribution on arterial or venous ECs might determine a different predisposition to cell activation or viral infection.

The glycocalyx dynamics between arterial and venous cells differ also upon EC activation. In agreement with previous studies^22,23,25,26,51–53^, we observed shedding of HS from the cell surface of arterial cells stimulated with normal human serum, TNFα, and LPS. Surprisingly, HS on venous cells was not affected (Figs. 2, 3), suggesting that HS on venous ECs is either less susceptible to shedding or protected against shedding. Shedding of the glycocalyx is largely mediated by matrix metalloproteinases (MMPs) which are upregulated in TNFα activated ECs^23^ and during sepsis, among other inflammatory conditions^54^. MMP activity is physiologically controlled by tissue inhibitors of matrix metalloproteinases (TIMPs) which also increase during inflammation^55^. Although unchanged expression of MMPs and/or maintained activity of TIMPs on venous but not arterial cells could account for the differential susceptibility to shedding, we did not observe changes in the expression of MMP2 and TIMP1 after activation with human serum, TNFα or LPS (data not shown). Further research is needed to analyze the expression of other MMPs and TIMPs and identify the mechanism behind the resistance of venous endothelial glycocalyx to shedding.

The persistence of HS on activated venous ECs implies that upon endothelial activation anti-inflammatory and anticoagulant properties are retained. This makes veins potentially more resistant to vascular damage than arteries. Indeed, we demonstrate that arterial cells have a HS-dependent anti-inflammatory and anticoagulant phenotype while the preserved HS on venous cells leads to variable functional outcomes when comparing different inflammatory stimuli. HS on venous cells protected against complement deposition and clotting only when TNFα or LPS was used for endothelial activation (Figs. 4, 5). This protective role of HS was not observed when a xenotransplantation setup was employed (Figs. 2, 3). This might be due to the simultaneous presence of multiple inflammatory stimuli during a xenotransplantation setting which elicit a strong inflammatory reaction. In fact, Wünsch et al.^56^ demonstrate that porcine arterial ECs produce a sustained intracellular calcium response to human serum compared to classical proinflammatory cytokines.

While differences in complement deposition on arterial and venous ECs were inflammatory-stimulus dependent, PBMC adhesion consistently occurred on both cell types (Figs. 3, 5). PBMC binding to venous cells correlated with cell surface expression of E-selectin (Supplementary Fig. S2) while interestingly, adhesion to arterial cells was also observed under non-inflammatory conditions where HS was simply removed from the cell surface by heparinase treatment. This suggests that shedding of HS from arterial cells is sufficient to induce leukocyte adhesion. Whether heparinase treatment on arterial cells exposes additional adhesion molecules other than E-selectin is unknown.

The importance of glycocalyx shedding for leukocyte adhesion has been a matter of debate. On one hand, an intact glycocalyx can shield off selectins and prevent leukocyte binding, as the thickness of the intact glycocalyx normally surpasses the length of adhesion molecules^57^. On the other hand, the glycocalyx sequesters chemokines to maintain elevated local concentrations and favor leukocyte recruitment^58^. Since leukocyte adhesion and extravasation usually occur in venules^59^, we suggest that venous ECs might actively maintain their glycocalyx to favor chemokine sequestration and mediate leukocyte recruitment during inflammation. Studies including chemokines as well as inflammatory stimuli would be needed to test this hypothesis. In contrast, HS shedding on arterial ECs might reduce the glycocalyx thickness and thereby expose adhesion molecules leading to increased leukocyte adhesion. Interestingly, McDonald et al.^17^ show that enzymatic removal of the glycocalyx from flow cultured arterial ECs even without an inflammatory stimulus increased ICAM-1 expression and leukocyte adhesion. This suggests that HS shedding could not only reduce the glycocalyx thickness but also lead to increased adhesion molecule expression on arterial ECs. However, for venous vessels, it was shown that binding of white blood cells occurred without glycocalyx shedding after an inflammatory stimulus^60^. This suggests that additional factors than glycocalyx thickness might play a role, one example being glycocalyx density. Platts et al.^61^ suggest that there is a “loosening” of the post-capillary venule glycocalyx after ischemia/reperfusion injury, allowing fluorescent dye to penetrate closer to the EC layer. This phenomenon could also apply to circulating leukocytes. A more “loose” glycocalyx on venous ECs could allow PBCMs to bind even with an intact glycocalyx. Our data on glycocalyx coverage shows lower coverage on venous than arterial cells, suggesting that the venous glycocalyx is less dense and possibly more permissive of leukocyte adhesion. On arterial ECs, where the glycocalyx coverage is denser, shedding is likely to expose adhesion molecules responsible for leukocyte binding.

Glycocalyx shedding is also known to induce a procoagulant EC phenotype. In fact, we observed that shedding of HS from arterial cells increases clot formation (Figs. 3, 5). Again, heparinase treatment was sufficient to induce this procoagulant EC phenotype. In contrast, on venous ECs, where HS was preserved, clotting was observed only after xenogeneic activation while it did not occur after stimulation with TNFα or LPS (Figs. 3, 5). Although proinflammatory cytokines and endotoxins are considered prothrombotic^62,63^, TNFα does not directly induce venous thrombosis^64,65^. TNFα receptor knock-out mice also develop thrombi after inferior vena cava ligation^65^. This suggests that changes in the surface expression of pro-thrombotic proteins, like tissue factor, rather than proinflammatory cytokines could drive venous thrombosis^66^.

Taken together our findings indicate that arterial cells are more susceptible to HS shedding. Indeed, removal of HS from the arterial cell surface is sufficient to initiate an inflammatory and thrombotic response. Instead, venous cells, on which HS is maintained, require further “hits” in addition to glycocalyx shedding to lose their anti-inflammatory and anticoagulant phenotype. Therefore, this suggests that venous ECs, compared to arterial ECs, are potentially more resistant to inflammatory insults. Distinct therapeutic approaches should be considered for different cell types: while replenishment of shed HS would be beneficial on arterial ECs, other approaches that don’t target glycocalyx recovery should be considered for venous ECs. Further research is needed to understand the mechanism behind venous HS shedding resistance.

While microfluidic systems are gaining popularity for in vitro research, it is important to mention that such systems can still differ from the in vivo situation. It should also be added that the research described above focused on macrovascular ECs, but microvascular- and organ-specific ECs should also be analyzed in order to expand the current knowledge on the heterogeneity of glycocalyx dynamics.

## Supplementary Information

Supplementary Figures.

## Data Availability

All relevant data are contained within the manuscript.
