# Supplementary material for: Inflammatory stimuli induce shedding of heparan sulfate from arterial but not venous porcine endothelial cells leading to differential proinflammatory and procoagulant responses
Source: Sci Rep. 2023 Mar 18;13:4483. doi: 10.1038/s41598-023-31396-z (PMC10024017; doi:10.1038/s41598-023-31396-z)
Supplement: Supplementary file 1 — Supplementary Figures. [file 41598_2023_31396_MOESM1_ESM.pdf]

Supplemental Material

Inflammatory stimuli induce shedding of heparan sulfate from arterial but not venous porcine endothelial cells leading to differential proinflammatory and procoagulant responses

Anastasia Milusev[1,2], Alain Despont[1], Jane Shaw[1], Robert Rieben[1] and Nicoletta Sorvillo[1*]

[1] Department for BioMedical Research (DBMR), University of Bern, Switzerland

[2] Graduate school for Cellular and Biomedical Sciences (GCB), University of Bern, Switzerland

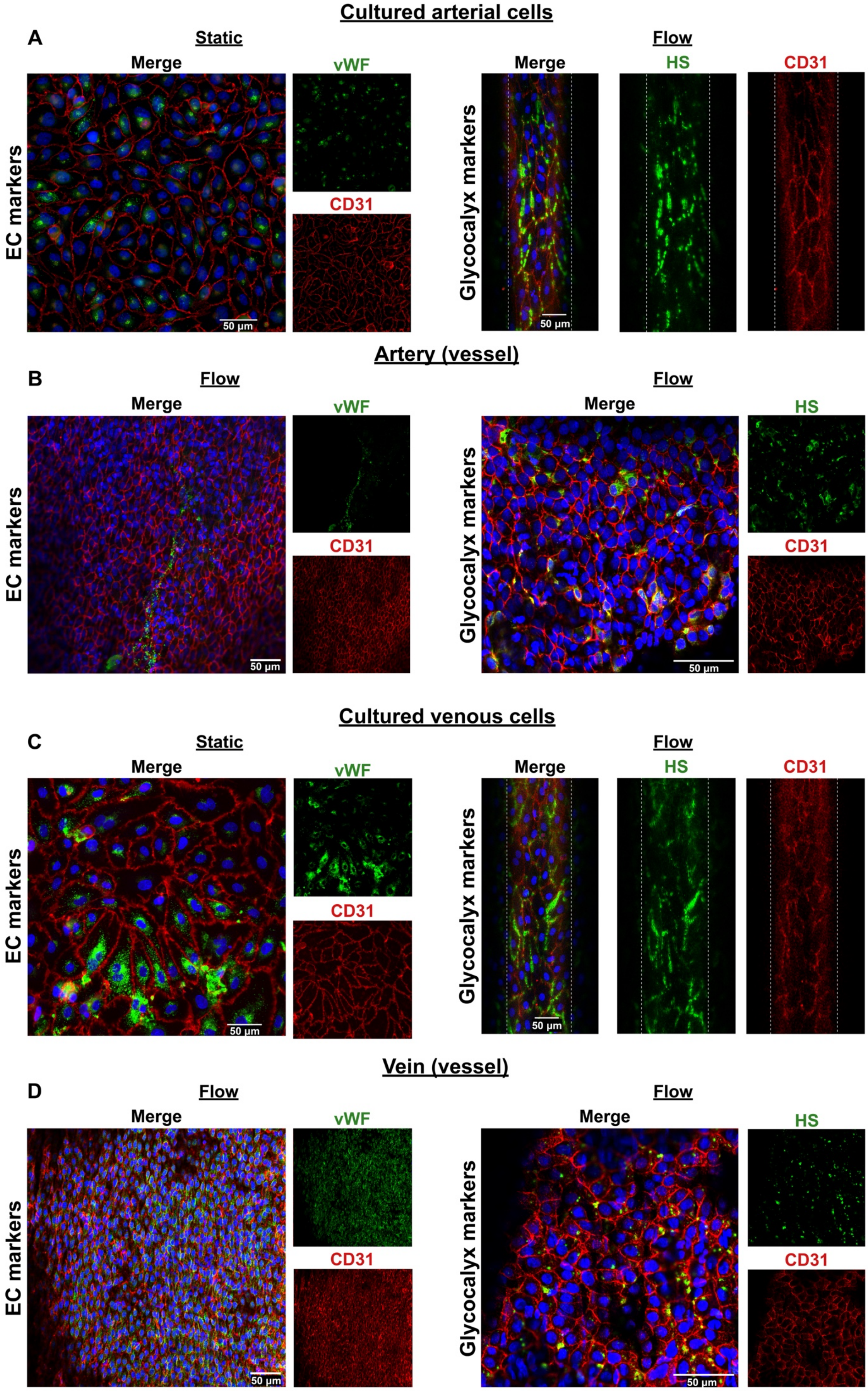
Cultured arterial cells
A
Static
Merge
vWF
CD31
EC markers
50 µm
Flow
Merge
HS
CD31
Glycocalyx markers
50 µm
Artery (vessel)
B
Flow
Merge
vWF
CD31
EC markers
50 µm
Flow
Merge
HS
CD31
Glycocalyx markers
50 µm
Cultured venous cells
C
Static
Merge
vWF
CD31
EC markers
50 µm
Flow
Merge
HS
CD31
Glycocalyx markers
50 µm
Vein (vessel)
D
Flow
Merge
vWF
CD31
EC markers
50 µm
Flow
Merge
HS
CD31
Glycocalyx markers
50 µm

Supplementary Figure S1: Cultured endothelial cells have a similar phenotype to endothelial cells within vessels. Representative images of cultured **(A)** arterial and **(C)** venous porcine endothelial cells or of freshly isolated porcine **(B)** thoracic aorta and **(D)** vena cava. Cells are stained for EC/glycocalyx marker von Willebrand Factor (vWF)/heparan sulfate (HS) in green and for CD31 in red. Nuclei are shown in blue (DAPI). Images were acquired with a confocal microscope (Zeiss LSM710). Representative images from three independent experiments are shown. Scale bar: 50μm.

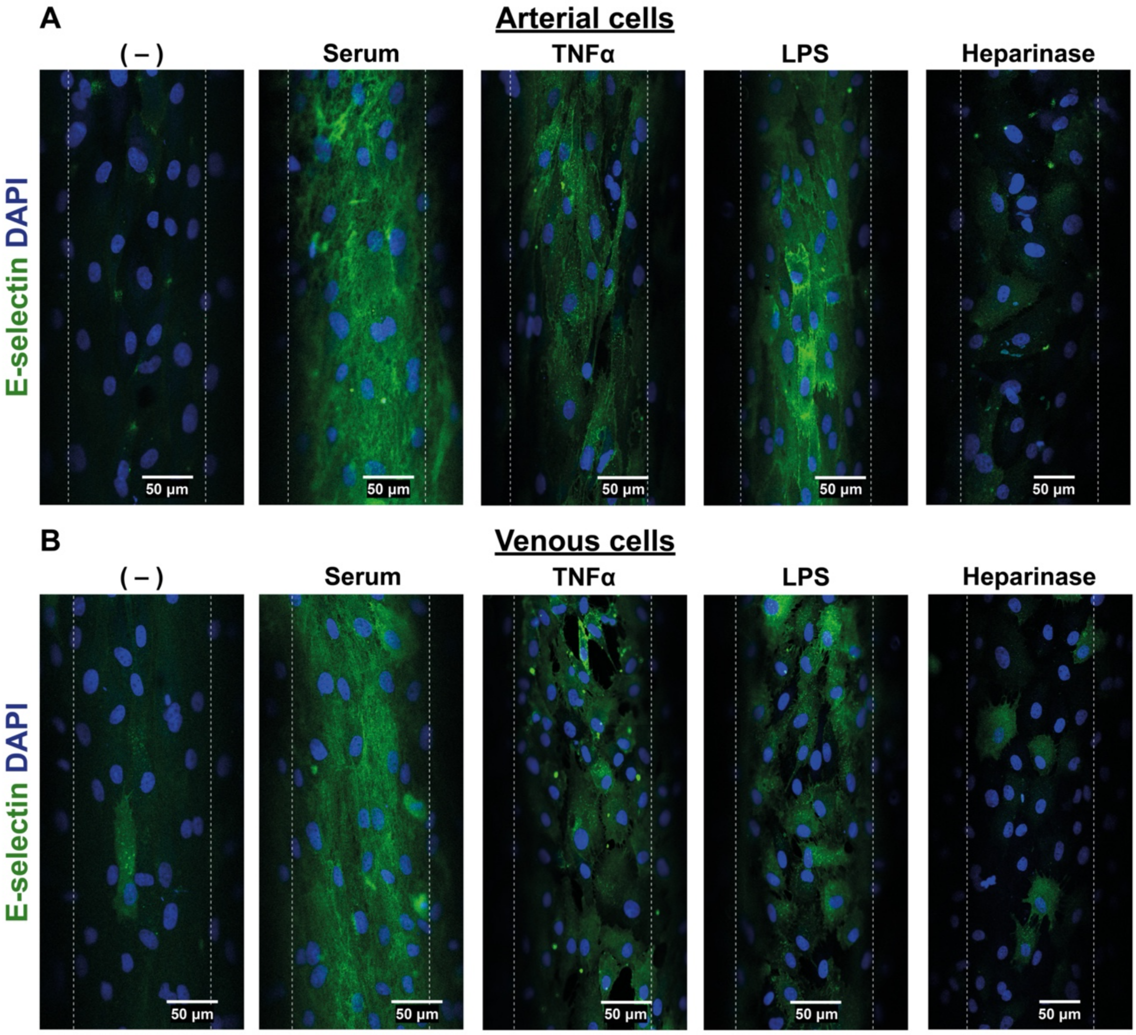

Supplementary Figure S2: E-selectin is expressed on arterial and venous endothelial cells after activation with human serum, TNFα or LPS. Representative images of microfluidic channels containing **(A)** arterial or **(B)** venous porcine endothelial cells were left untreated (-) or perfused with either 10% normal human serum (Serum), 100ng/ml TNFα, 100ug/ml LPS or 5U/ml heparinase (Heparinase). E-selectin is shown in green and nuclei in blue (DAPI). Images were acquired with a confocal microscope (Zeiss LSM980). Representative images from three independent experiments are shown. Scale bar: 50μm.

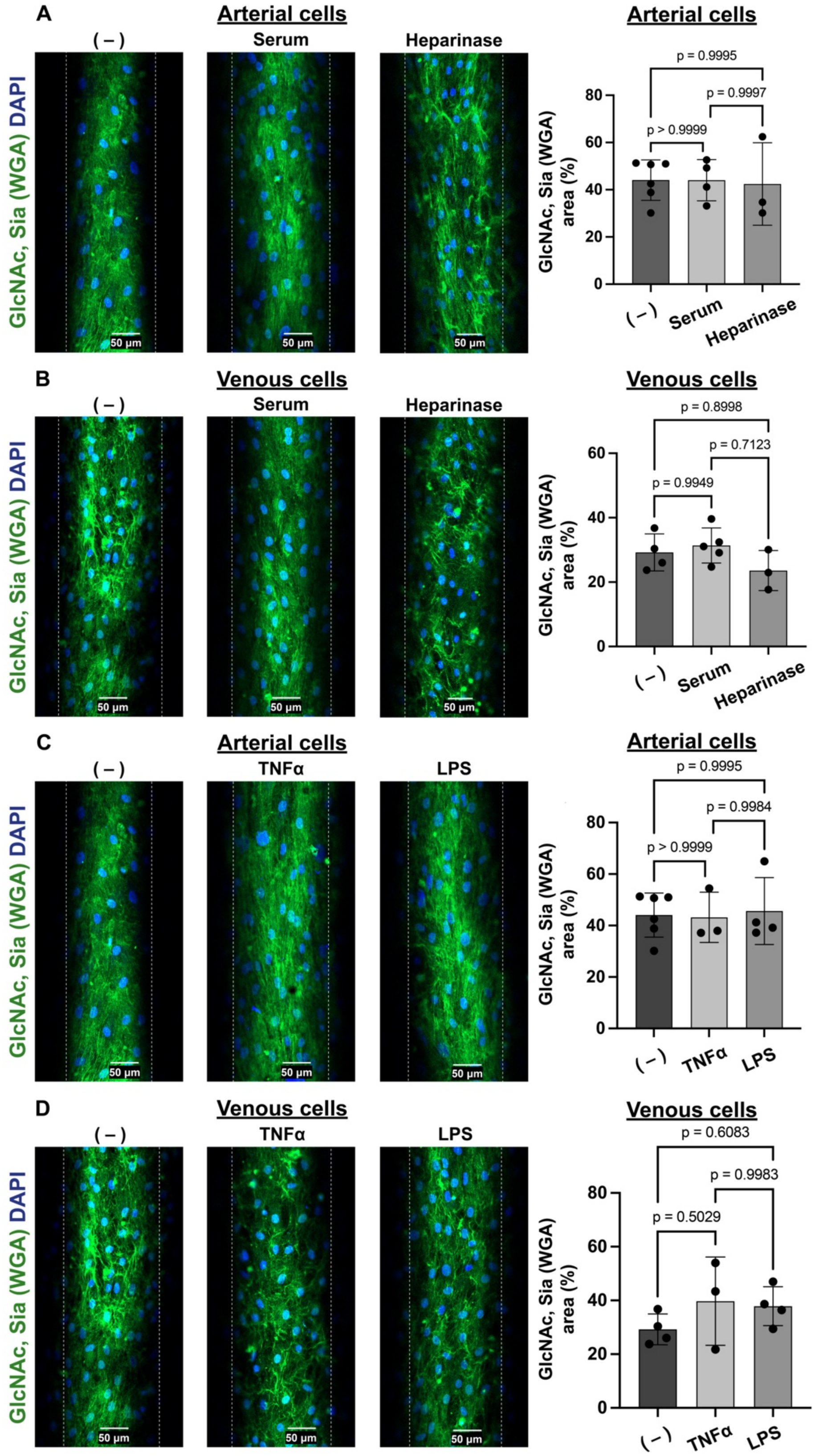

Supplementary Figure S3: N-Acetylglucosamine and sialic acid on arterial and venous endothelial cells after activation with human serum, TNFα or LPS. Representative images of microfluidic channels containing **(A, C)** arterial or **(B, D)**

venous porcine endothelial cells left untreated (-) or perfused with either 10% normal human serum (Serum), 5U/ml heparinase (Heparinase), 100ng/ml TNFα or 100μg/ml LPS. N-Acetylglucosamine (GlcNAc) and sialic acid (Sia) are shown in green and nuclei in blue (DAPI). Cell surface coverage of GlcNAc/Sia was quantified for each image (4 images/condition/experiment) as the percentage of area positive for GlcNAc/Sia and normalized for the total number of cells/image. Data was analyzed using Fiji software. All images were acquired with a Zeiss LSM980 confocal microscope. Data are from three or more independent experiments. One-way ANOVA with multiple comparisons was used for statistical analysis. Scale bar: 50μm

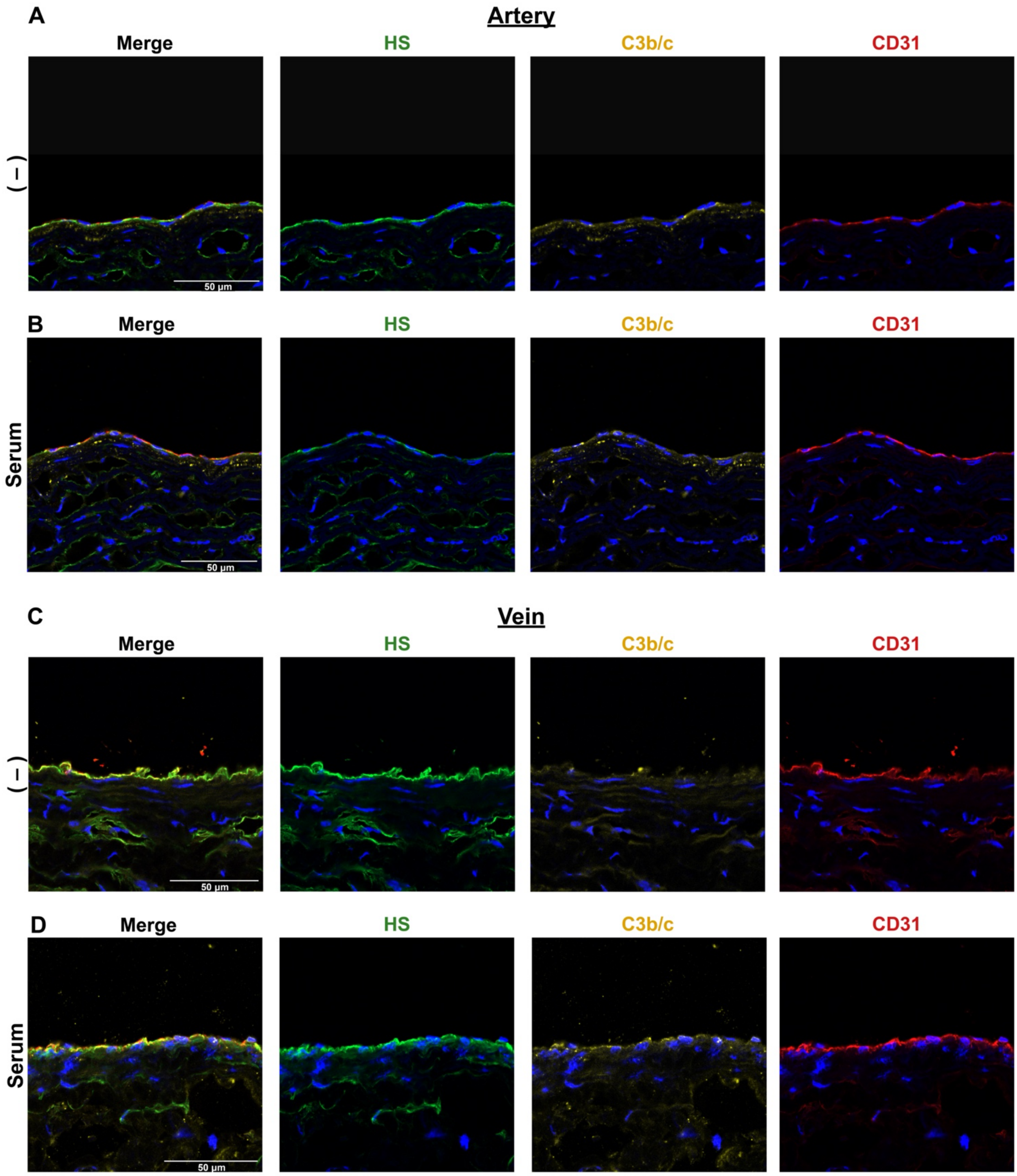

Supplementary Figure S4: Shedding of heparan sulfate and complement deposition on freshly isolated porcine vessels. Representative images of heparan sulfate (HS) expression on porcine **(A, B)** thoracic aorta and **(C, D)** vena cava. Vessels were **(A, C)** left untreated (-) or **(B, D)** treated with 10% normal human serum (Serum)

and stained for heparan sulfate (HS) in green, complement C3b/c in yellow and CD31 in red. Nuclei are shown in blue (DAPI). All images were acquired with a Zeiss LSM980 confocal microscope. Scale bar: 50μm

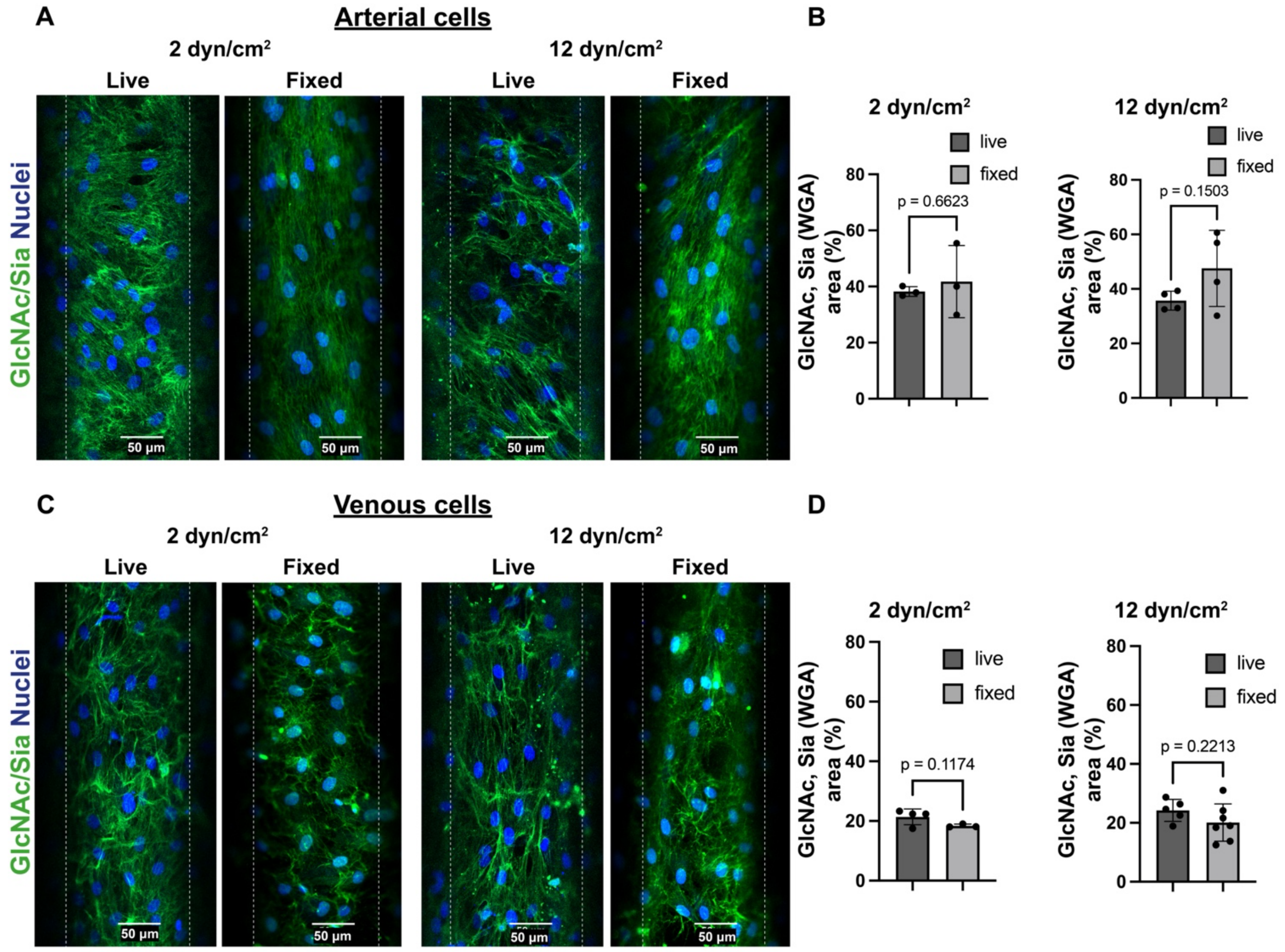

Supplementary Figure S5: Coverage of N-Acetylglucosamine and Sialic acid on live and fixed arterial and venous porcine endothelial cells under different shear stress conditions. Representative images of microfluidic channels containing arterial **(A)** and venous **(C)** porcine endothelial cells cultured at a low (2 dyn/cm^2) or high (12 dyn/cm^2) shear stress for 72h. To visualize N-Acetylglucosamine and Sialic acid (GlcNAc/Sia) live cells were stained under flow with WGA-Lectin (green) and Hoechst nuclear stain (blue) whereas fixed cells were first fixed with 4% formaldehyde and then stained with WGA-Lectin (green) and DAPI to visualize nuclei (blue). All images were acquired with a Zeiss LSM980 confocal microscope. Scale bar: 50 μm **(B, D)** GlcNAc/Sia coverage was quantified for each image (3-4 images/condition/ experiment) as the percentage of area positive for GlcNAc/Sia and normalized for the total number of cells/image. Student's t-test was used for statistical analysis

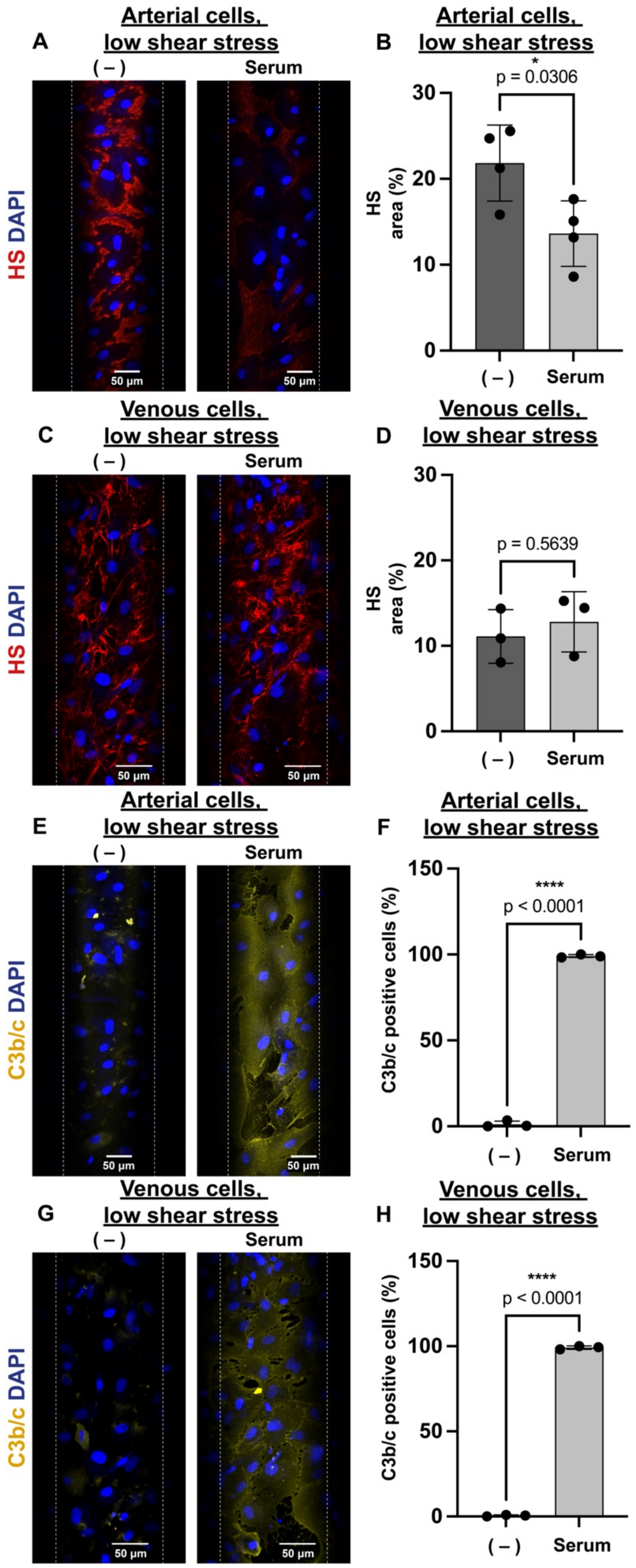

Supplementary Figure S6: Persistence of heparan sulfate on xenogeneic activated venous endothelial cells at low shear stress does not prevent C3b/c

deposition. Representative images of microfluidic channels containing **(A, E)** arterial and **(C, G)** venous porcine endothelial cells cultured at a low shear stress (2 dyn/cm^2) and left untreated (-) or activated with 10% normal human serum (Serum). **(A, C)** Cells are stained for heparan sulfate (HS) in red and nuclei are shown in blue (DAPI). **(B, D)** HS coverage was quantified for each image (4 images/ condition/experiment) as the percentage of area positive for HS and normalized for the total number of cells/image. **(E, G)** Representative images of complement C3b/c deposition on the cell surface of cells left untreated (-) or activated with 10% normal human serum (Serum). Complement C3b/c is shown in yellow and nuclei in blue (DAPI). **(F, H)** Complement deposition was quantified as percentage of C3b/c positive cells/total number of cells (4 images/condition/experiment). Images were acquired with a Zeiss LSM980 or LSM710 confocal microscope. Data are from three or more independent experiments. Student's t-test was used for statistical analysis. Scale bar 50μm.

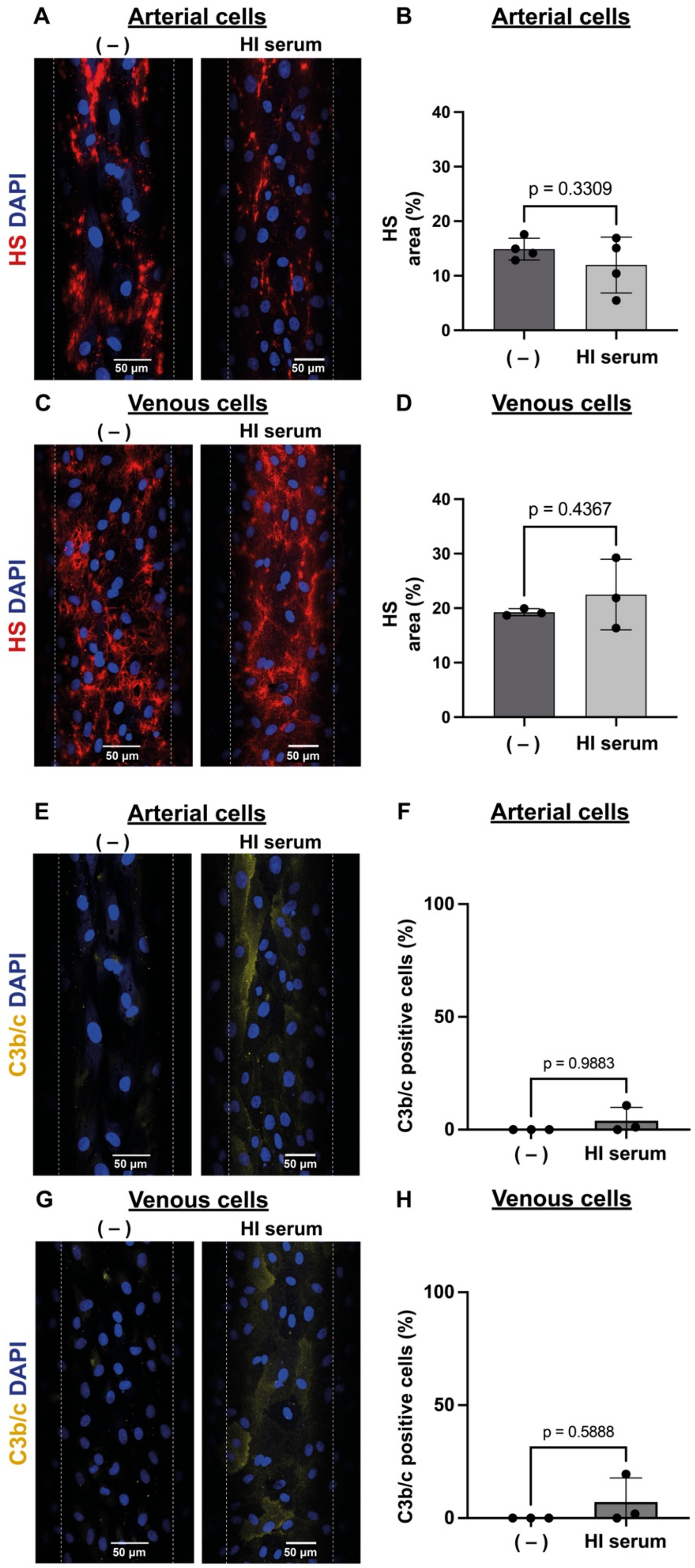

Supplementary Figure S7: Xenogeneic activation with heat inactivated human serum prevents heparan sulfate shedding and complement deposition on both

arterial and venous endothelial cells. (A, C) Representative images of cells left untreated (-) or activated with 10% heat inactivated normal human serum (HI serum). Heparan sulfate (HS) is shown in red and nuclei in blue (DAPI). Scale bar: 50μm **(B, D)** Shedding of HS was quantified for each image (4 images/condition/experiment) as the percentage of area positive for HS and normalized for the total number of cells/image. **(E, G)** Representative images of complement C3b/c deposition on the cell surface of cells left untreated (-) or activated with 10% heat inactivated normal human serum (HI serum). Complement C3b/c is shown in yellow and nuclei in blue (DAPI). **(F, H)** C3b/c deposition was measured as the percentage of C3b/c positive cells/total number of cells (4 images/condition/experiment). Data was quantified with Fiji software. All images were acquired with a Zeiss LSM980 confocal microscope. Data are from three or more independent experiments. One-way ANOVA with multiple comparisons was used for statistical analysis.
